# Selections

**Published:** 1892-04

**Authors:** 


### Selections.

### A CASE OF COCAINE-POISONING.

On September 23 a lady, the wife of a railroad conductor, called to have the right superior first molar extracted. The crown was gone ; roots and socket very sore from peridental inflammation. A twelve-per-cent. solution of hydrochlorate cocaine was applied to the surface of the gum twice at an interval of five minutes; none used hypodermically. The operation of extraction gave great pain, notwithstanding the use of the cocaine. No bad effects were noticed from the cocaine used.

She had an upper left wisdom tooth to be extracted, but postponed it for another sitting. On the 2d of October (about a week after this) she came accompanied by her husband to have the wisdom tooth extracted. She was very nervous and fearful about the expected operation, and, in fact, was almost in a state of nervous prostration from worry, pain, and loss of sleep, as the tooth had been aching very severely for several days.

This time a twelve-per-cent. solution of the cocaine was applied to the gums as before, after which a four-per-cent. solution of cocaine was injected first on the outside or buccal surface of the tooth and then on the palatine surface, passing the needle deeply into the tissues alongside the tooth,—about one-half inch, I think.

Almost immediately after removing the needle—the last injection—symptoms of cocaine poisoning began to show themselves. The first effects noticeable were those of a person just in the act of fainting, followed in quick succession by violent twitching, convulsive movements of the eyeballs, the general tendency of which seemed to be upward, under the upper lids, crossing of the eyes, etc. The respiration became very short and quick, as if a paralysis of the lungs were coming on; the pulse became very rapid, weak, and thready,—in fact, was at times almost imperceptible. One peculiarity of her condition was, that for short intervals the violent and alarming symptoms would pass away and she would appear to be reacting, but only momentarily, for she would relapse again into more violent paroxysms than before. During this time she was apparently unconscious. There is reason to believe that she was entirely so.

Of course prompt measures were taken to restore her. The chair was thrown back until her head was brought below a horizontal line, cold, wet towels applied to her head and face, her clothing loosened, and a physician sent for. As soon as it could be procured, which, fortunately, was in a very few minutes, she was given a drink of hot, strong coffee; the effect of which was like magic, for her alarming symptoms subsided at once. Her breathing became easier, her pulse stronger, she became conscious,—in fact, in a few minutes she was out of danger. She was very much prostrated, being so weak that it was several hours before she could leave the chair. The physician gave her a dose of digitalis before he left, and she was also given brandy afterwards, but the remedy that relieved her was the coffee.

Dr. T. C. Edwards,
*Texas Dental Journal.*

### LOCAL ANÆSTHESIA BY AQUAPUNCTURE.

Dr. C. Schleich is convinced, from experiments upon himself and his assistants, that local anæsthesia, sufficient for minor surgical operations, can be obtained by using a spray of ether in liquid vaseline (4 to 1), followed by hypodermic injections of pure sterilized water. The needle is introduced at first, in the usual way, below the surface of the skin and then parallel to it, forming at the point of injection an œdema of the skin. In the extent of this œdema the skin is absolutely insensible.

M. Schleich has, by means of this procedure, made, without pain, in a case of anthrax, two crucial incisions, eight centimetres (three inches) long, and excised and scraped out the dead tissue.—*Les Nouveaux Remèdes*, October 8, 1891, *The Satellite.*

---

### DISINFECTOL.

This substance is analogous to creolin and lysol. It is a brownish-black, oily liquid, having a density of 1.086. It has an alkaline reaction, and contains hydrocarbons, soaps of resin, and combinations of soda and carbolic acid. It has, it is said, very energetic disinfectant properties. It is used in the form of an emulsion, containing from two to five per cent.—*Journal de Médecine, de Chirurgie, et de Pharmacologie*, November 20, 1891, *The Satellite.*

---

### THERAPEUTICS AND TOXICOLOGY.

### IODO-NAPHTHOL-BETA—A NEW ANTISEPTIC.

M. Braille (*Répertoire de Pharmacie*) has prepared with naphthol-beta and iodine a new antiseptic resembling aristol, to which he has given the name iodo-naphthol-beta, or naphthol-beta diiodide. It is an inodorous, tasteless, greenish-yellow powder, insoluble in water, slightly soluble in ether, freely soluble in chloroform, and almost insoluble in alcohol or acetic acid.—*La Médecine Moderne*, November 19, 1891, *The Satellite.*

